# Expression of ADAM Proteases in Bladder Cancer Patients with BCG Failure: A Pilot Study

**DOI:** 10.3390/jcm10040764

**Published:** 2021-02-14

**Authors:** Renate Pichler, Andrea Katharina Lindner, Georg Schäfer, Gennadi Tulchiner, Nina Staudacher, Martin Mayr, Eva Comperat, Jacob J. Orme, Gert Schachtner, Martin Thurnher

**Affiliations:** 1 Department of Urology, Medical University of Innsbruck, 6020 Innsbruck, Austria; andrea.lindner@i-med.ac.at (A.K.L.); Gennadi.Tulchiner@i-med.ac.at (G.T.); Nina.Staudacher@i-med.ac.at (N.S.); martin.t.mayr@student.i-med.ac.at (M.M.); Gert.Schachtner@tirol-kliniken.at (G.S.); 2 Department of Pathology, Medical University of Innsbruck, 6020 Innsbruck, Austria; georg.schaefer@i-med.ac.at; 3 Department of Pathology, Medical University of Vienna, 1090 Vienna, Austria; eva.comperat@meduniwien.ac.at; 4 Division of Medical Oncology, Mayo Clinic, Rochester, MN 55902, USA; orme.jacob@mayo.edu; 5 Immunotherapy Unit, Department of Urology, Medical University of Innsbruck, 6020 Innsbruck, Austria

**Keywords:** bacillus calmette guéri (BCG) failure, bladder cancer, a disintegrin and metalloproteinase (ADAM) proteases, soluble PD-L1, PD-L1 biomarkers, checkpoint inhibition, immunotherapy

## Abstract

Although Bacillus Calmette Guérin (BCG) remains a mainstay of adjuvant treatment in high-risk, non-muscle-invasive bladder cancer, BCG failure occurs in up to 40% of patients, with radical cystectomy (RC) as the inevitable therapeutic consequence. Current data suggest that PD-L1 immunosuppressive signaling is responsible for BCG failure, supporting the therapeutic rationale of combining checkpoint inhibitors with BCG. To address the immune cascade in 19 RC specimens obtained after BCG failure, we applied a small immunohistochemical (IHC) panel consisting of selected markers (PD-L1, GATA-3, a disintegrin and metalloproteinase (ADAM) proteases, IL-10/IL-10R). A modified quick score was used for IHC semi-quantification of these markers in tumor cells (TC) and immune cells (IC) within two different regions: muscle-invasive bladder cancer (MIBC) and primary/concurrent carcinoma in situ (CIS). Contrary to expectation, PD-L1 was consistently low, irrespective of tumor region and cell type. Intriguingly, expression of ADAM17, which has been reported to release membrane-bound PD-L1, was high in both tumor regions and cell types. Moreover, expression of GATA3, IL-10, and IL-10R was also increased, indicative of a generally immunosuppressive tumor microenvironment in BCG failure. ADAM10 expression was associated with advanced tumor disease at RC. Our findings raise the possibility that ADAM proteases may cleave PD-L1 from the surface of bladder TC and possibly also from IC. Therefore, IHC assessment of PD-L1 expression seems to be insufficient and should be supplemented by ADAM10/17 in patients with BCG failure.

## 1. Introduction

Bladder cancer is among the most immunogenic tumors. Morales and coworkers demonstrated in 1976 that bladder cancer responds to the intravesical instillation of Bacillus Calmette Guérin (BCG) [1], a live attenuated vaccine originally developed against tuberculosis. Since then, BCG has been the gold-standard adjuvant immunotherapy for patients with high-risk non-muscle-invasive bladder cancer (NMIBC). While the precise immunologic mechanism that generates protective antitumor immunity in bladder cancer remains unclear, previous work has, for instance, shown that BCG causes the maturation of immunogenic dendritic cells, which promote adaptive immunity [2,3]. In addition, BCG has been shown to induce a form of innate immune memory, referred to as trained immunity [4,5]. Innate immune cells such as macrophages can be primed, i.e., trained first by a challenge with BCG, resulting in an augmented cytokine response to secondary stimulation with BCG or even with an unrelated stimulus. The increased responsiveness of trained macrophages is due to BCG-induced functional reprogramming caused by metabolic rewiring [6]. At least in mice, BCG-induced long-term innate immune memory occurred at the level of hematopoietic stem cells and multipotent progenitors in the bone marrow and was mediated by IFN-γ [7]. These observations indicated that access of BCG to the bone marrow was required and that BCG locally enhanced myelopoiesis, generating trained macrophages that confer increased immune protection.

According to the most recent European Association of Urology (EAU) guideline for high-risk NMIBC, the intravesical BCG treatment schedule comprises a 6-week induction course with weekly instillations, followed by a 3-year BCG maintenance schedule [8].

Despite the considerable efficacy of BCG treatment, BCG failure is observed in up to 40% of patients with high-risk NMIBC, with radial cystectomy (RC) becoming the inevitable therapeutic consequence [9]. BCG failure thus represents a challenge in clinical practice and calls urgently for markers predicting response to BCG therapy and for improved treatment regimens for the non-responsive patient population. We previously described pre-therapeutic intratumoral expression of the transcription factor GATA3 associated with an increased Th1 functional phenotype in BCG responders compared to BCG failure [10]. The recent finding that baseline tumor PD-L1 expression may predict an unfavorable response to BCG [11] suggested that PD-1/PD-L1 immunosuppressive signaling may be responsible for BCG failure. PD-L1 has also been shown to be upregulated during BCG therapy, both in vitro and in vivo [12]. Additionally, PD-L1 expression was associated with high-grade bladder tumors, representing the highest expression levels in carcinoma in situ (CIS) with 45% [13]. Finally, increased expression of PD-1 was noticed in RC specimens from patients with previous BCG therapy [14], supporting the therapeutic rationale of targeting checkpoint inhibitors with BCG in ongoing clinical trials (NCT03528694; NCT03711032). The first results of the single-arm phase II KEYNOTE-057 trial of pembrolizumab in patients with BCG-nonresponsive high-risk NMIBC (or ineligible for RC) are encouraging, presenting complete responses in 38.8% at 3 months [15].

However, results about the effect of intravesical BCG on PD-L1 expression and reproducibility of PD-L1 following BCG failure are controversial. For example, a significant increase of PD-L1 gene expression on RNA or protein levels between pre-treatment and post-treatment tumor samples could not be observed [11]. Moreover, neither pre-treatment nor post-treatment PD-L1 expression analysis appeared to have clinical prognostic utility for high-grade NMIBC treated with BCG [16]. Additionally, there was no significant association between PD-L1 positivity and tumor recurrence after BCG induction [17].

To better understand the immunologic mechanisms underlying BCG failure, we have examined for the first time the expression of ADAM17, a disintegrin and metalloproteinase (ADAM) family member in BCG-nonresponsive bladder cancer. ADAM17 has recently been shown to proteolytically cleave (shed) membrane-bound PD-L1, causing the release of soluble PD-L1 ectodomains [18].

## 2. Materials and Methods

### 2.1. Patients and Treatment

This study was approved by the local ethics committee (study number 1006/2017). Medical records of patients with BCG failure, thus undergoing consecutive RC at our department, were reviewed retrospectively from our cystectomy database. In all patients, intravesical BCG induction therapy was given due to high-risk NMIBC according to the European Organization for Research and Treatment of Cancer (EORTC) scoring system [19] in a 6-weekly schedule once a week, followed by maintenance therapy for 3 years (3-weekly once a week at 3, 6, 9, 12, 18, 24, 30, and 36 months) and uro-oncological follow-up according to the European Association of Urology (EAU) guidelines [8]. All patients completed six series of BCG induction. A histologically confirmed upstaging to muscle-invasive bladder cancer detected during follow-up (=progression) or a high-grade relapse (=high-grade recurrence) after BCG induction/during BCG maintenance was defined as BCG failure. At BCG failure, all patients underwent immediate RC with extended pelvic lymphadenectomy and urinary diversion. According to our institutional practice and current literature showing worse survival in patients with secondary muscle-invasive bladder cancer (MIBC) treated with neoadjuvant chemotherapy compared with cystectomy alone [20], no patient with secondary MIBC (*n* = 8) received neoadjuvant chemotherapy. Adjuvant cisplatin-based chemotherapy (4 cycles of gemcitabine 1000 mg/m2 on days 1, 8, and 15, and cisplatin 70 mg/m2 on day 2; 1 cycle = 28 days) was applied in 3 (15.8%) patients with extravesical disease and concomitant pN+ status.

### 2.2. Tumor Samples

All radical cystectomy (RC) specimens were re-reviewed in regard to diagnosis, tumor grade (WHO 1973 and 2004), and stage (TNM 2009) by one experienced uropathologist (G.S.) for study purposes. One representative tumor block of every case was selected for further immunohistochemical (IHC) analysis. Consecutive slides were used to compare the same field of view in a given case. Histopathological specimens included muscle-invasive bladder cancers (*n* = 14) with concomitant carcinoma in situ (CIS) tumors (*n* = 10) as well as primary CIS lesions (*n* = 5). Therefore, the localization patterns and expression levels of various markers on tumor cells and immune cells were analyzed in two different regions: at the invasive front and in the case of CIS, the underlying lamina propria and the overlying neoplastic urothelium were used for analysis.

### 2.3. Immunohistochemistry (IHC)

The following antibody panel was used: GATA3 (Monoclonal Mouse Anti-Human GATA3, Clone L50-823, prediluted, Roche Nr.7107749001), PD-L1 (Monoclonal Rabbit Anti-Human PD-L1 clone SP263, prediluted, Roche Nr.7494190001), ADAM17 (Polyclonal Rabbit Anti-Human Adam17, dilution 1:100, Abcam ab2051), ADAM10 (Polyclonal Rabbit Anti-Human Adam10, dilution 1:500, Abcam ab1997), IL-10 (Polyclonal Rabbit Anti-Human IL-10, dilution 1:100, Abcam ab84843), and IL-10R (Polyclonal Rabbit Anti-Human IL-10RA, dilution 1:30, Abcam ab197666).

**Figure 1:**
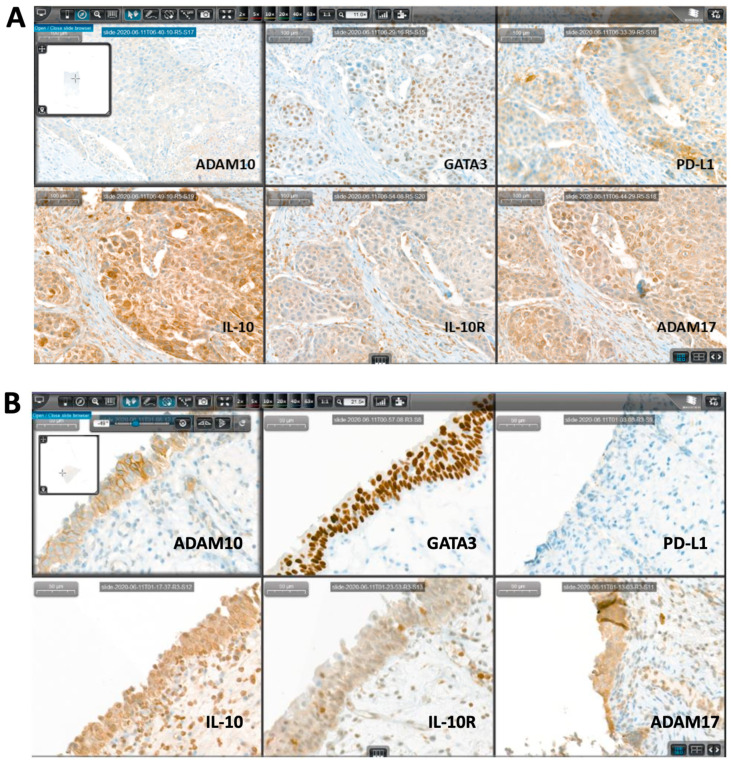
Representative immunohistochemical (IHC) figures of the complete IHC panel stained on consecutive sections using CaseViewer digital microscopy for systematic analysis. The presented cases show a pT2b urothelial carcinoma (**A**) and a primary carcinoma in situ (CIS) (**B**) on radial cystectomy (RC) specimen.

For testing and positive control of all markers, human tissue corresponding to the manufacturer’s recommendations were used. For the negative control, one slide of each RC specimen obtained after BCG failure was incubated without primary antibody. Representative stains and quantifications for PD-L1, GATA3, ADAM17, ADAM10, IL-10, and its receptor of four selected patients are shown in Figure 1 and in Supplementary Figure S1.

Staining was performed using an automated immunostainer (BenchMark ULTRA, Ventana Medical Systems/Roche, Tucson, AZ, USA). Briefly, formalin-fixed, paraffin-embedded (FFPE) tissue sections were cut in widths of 1.5 μM. After deparaffinization, the slides were treated with cell conditioning reagent 1 (CC1, Roche Nr.950-124) for antigen retrieval. All primary antibodies were incubated for 32 min, except IL-10 for 2 h, at 37 °C. The Ultra View DAB Detection Kit (Roche Nr.760-500) and OptiView DAB IHC Detection Kit (Roche Nr.760-700) for PD-L1, respectively, were used for visualization in accordance with the manufacturer’s recommendations. Finally, slides were washed in distilled water, counterstained with hematoxylin (12 min) and bluing reagent (4 min), dehydrated in a descending order of alcohols, cleared in xylene, and cover-slipped with Tissue-Tek mounting medium (Sakura Finetek, Tokyo, Japan).

### 2.4. Quantification of Tumor Cells (TC) and Immune Cells (IC)

Stained slides were digitally scanned by a Pannoramic 250 Flash III scanning system (3DHISTECH, 1141 Budapest, Hungary), and for each case, the complete IHC panel, which was stained on consecutive sections, was aligned using CaseViewer digital microscopy software (3DHISTECH, 1141 Budapest, Hungary) for systematic analysis by an experienced uropathologist (G.S.). Representative stains for two selected patients (pT2b urothelial carcinoma and primary CIS) demonstrating CaseViewer digital microscopy are shown in Figure 1.

A modified quick score [21] combining a staining intensity score (0–3, 0: no/1: weak/2: moderate/3: strong staining) and percentage of stained cells score (0–4, 0: 0%/1: 1–4%/2: 5–9%/3: 10–49%/4: 50–100%) was used for IHC semi-quantification of tumor cells (TC) and immune cells (IC) within two different regions (MIBC and carcinoma in situ). The product of both scores (combination score = % score (0–4) x intensity score (0–3)) was used to indicate the level of protein expression. The protein expression was considered negative if the combination score was ≤3 and positive if 4 or greater and was used for further statistical analysis in Table 1.

### 2.5. Statistics

IHC expression levels between tumor regions and cell types were compared with Fisher’s exact tests, Mann–Whitney U tests, and independent Kruskal–Wallis tests. Friedman’s two-way analysis of variance (ANOVA) and Wilcoxon’s signed-rank tests were used for comparison of paired groups. Correlations between parameters were assessed with Spearman’s ρ correlation coefficient (rs). A significance level of α = 0.05 (two-tailed) was applied for all *p*-values. SPSS, version 22.0 (SPPS Inc., Chicago, IL, USA) was used for statistical analysis. Graphic diagrams were produced with GraphPad PrismTM6, version 9.0.1 (GraphPad Software Inc., La Jolla, CA, USA). All values were presented as mean ± standard error of the mean (SEM).

## 3. Results

### 3.1. Baseline Characteristics of Patients with BCG Failure

A total of 19 (14 male and 5 female) patients aged on average 68 (SD ± 12.4; median: 77; range: 37–81) years were included in this study. BCG induction and maintenance were applied in all patients due to primary high-risk NMIBC. Detrusor muscle was present in all histopathological specimens of primary transurethral resection of the bladder (TURB). A second resection was performed in all high-risk NMIBC patients (except primary CIS) prior to BCG induction. In all patients, BCG failure was histologically confirmed by TURB followed by RC. The mean time interval between BCG induction and BCG failure was 16 (range: 3–21) months. A detailed overview of histopathological features concerning primary TURB (prior BCG), TURB at BCG failure, and finally, RC specimens are described in Table 2. Pure urothelial carcinoma was confirmed in 18 patients, whereas one patient showed urothelial carcinoma with partial squamous differentiation.

**Figure 2:**
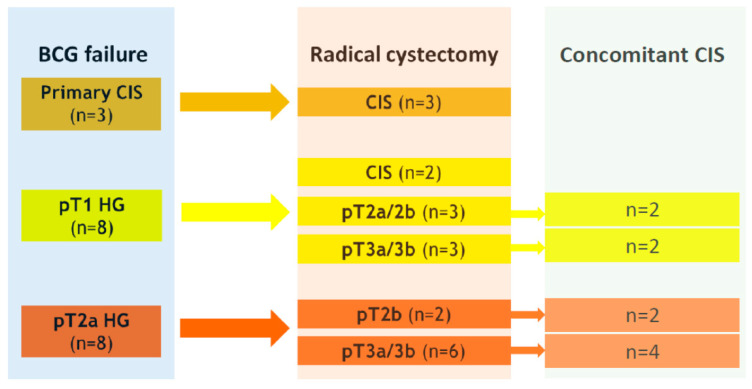
Schematic illustration of histopathologic associations between BCG failure and radical cystectomy.

An upstaging to at least muscle-invasive disease in final RC specimens occurred in six (75%) of eight pT1 HG tumors at BCG failure, respectively (Figure 2). Overall, five patients (28.3%) had positive pathological lymph node status (pN+), and nine patients (47.3%) confirmed extravesical disease at final histology on RC specimens.

### 3.2. Expression of ADAM Proteases and Lack of PD-L1 Expression in RC Specimens Obtained from Patients with BCG Failure

**Figure 3:**
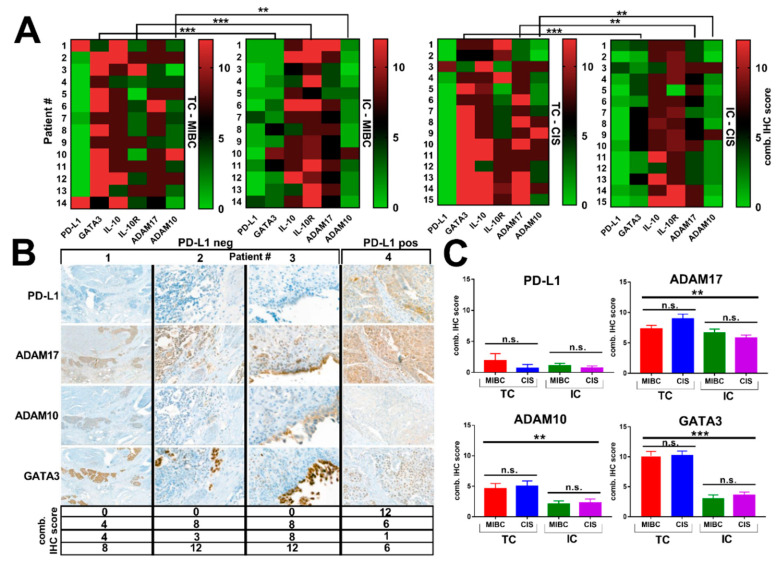
(**A**) Heat mapping with continuous green-black-red shading. Heat maps from individual patients (labeled 1 to 14 or 1 to 15) are arranged from top to bottom left based on increasing IHC combined score (0–12) of PD-L1, GATA3, IL-10, IL-10R, ADAM17, and ADAM10. Barlines show the direct comparison between TC and IC within MIBC and CIS. * *p* < 0.05; ** *p* < 0.01; *** *p* < 0.001 according to Mann–Whitney *U* test. (**B**) Representative IHC images of PD-L1, ADAM17, ADAM10, and GATA3 within MIBC for four selected patients, presenting three patients with PD-L1 negative status (#1–3, pure urothelial carcinoma, comb. score of 0 in all patients) and one patient (#4) with positive PD-L1 expression (combined score of 12, urothelial carcinoma with squamous differentiation). Final histology at RC specimens confirmed pT3b + CIS N0R0 (patient #1), pT3a N2R0 (patient #2), pT1 HG + CIS N0R0 (patient #3), and pT3b N1R0 (patient #4). Irrespective of PD-L1 status, patients display GATA3 and simultaneous ADAM17 expression. (**C**) Mean IHC combined scores for PD-L1, ADAM17, ADAM10, and GATA3 assessed for both tumor regions (MIBC and CIS) and both cell types (TC and IC) are shown. Statistically significant differences of expression patterns were confirmed for GATA3, ADAM17, and ADAM10. Data represent mean ± SEM (* *p* < 0.05; ** *p* < 0.01; *** *p* < 0.001 according to independent samples Kruskal–Wallis test and Mann–Whitney *U* test).

Interestingly, we did not detect PD-L1 expression in the majority (78.9%) of tumor specimens from patients with BCG failure (Figure 3A,B). PD-L1 was consistently low, irrespective of tumor region and cell type, and most importantly, PD-L1 was not increased in CIS lesions compared to MIBC (Figure 3C). Intriguingly, we identified consistently increased TC and IC expression of ADAM17 within the MIBC, whereas its expression in CIS was significantly higher in TC than IC (*p* = 0.003) (Figure 3A). Moreover, ADAM17 expression was high in both tumor regions and cell types (Figure 3C). Compared to ADAM17, ADAM10 expression was significantly lower in both tumor regions, with higher levels in TC compared to IC within MIBC (*p* = 0.004) and CIS (*p* = 0.007) (Figure 3A,C). Overall, significant differences in the expression levels of IHC markers between TC and IC were assessed for GATA3 (*p* < 0.001), IL-10R (*p* < 0.0001), and ADAM10 (*p* = 0.004) within MIBC, and for GATA3 (*p* < 0.0001), ADAM17 (*p* = 0.003), and ADAM10 (*p* = 0.007) within CIS (Figure 3A). BCG-failure tumors were homogeneously GATA3 positive, irrespective of tumor region (MIBC and CIS), but with significantly increased expression levels in TC compared with IC within MIBC (*p* < 0.0001) and CIS (*p* < 0.0001) (Figure 3A,C).

Moreover, IL-10 expression was also increased, with similar expression levels in TC and IC on both tumor regions (mean combined score, TC vs. IC: 8.8 vs. 7.8, *p* = 0.587 for MIBC; 9.3 vs. 8.3, *p* = 0.333 for CIS) (Supplementary Figure S1). IL-10R expression was significantly higher in IC within MIBC (TC vs. IC, mean combined score: 4.9 vs. 10.1; *p* < 0.0001), but not in CIS compared to TC (*p* = 0.102) (Supplementary Figure S1).

### 3.3. Significant Correlations between PD-L1 and GATA3 as well as ADAM17 and IL-10R

**Figure 4:**
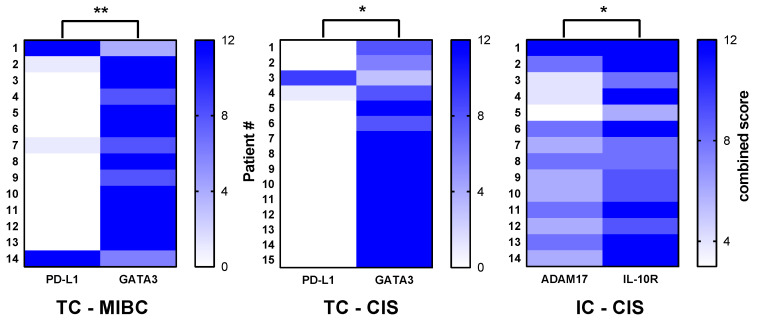
Spearman’s correlation analysis. Negative association between PD-L1 and GATA3 expression in tumor cells (TC) within both tumor regions, and a positive correlation between ADAM17 and IL-10R expression in immune cells (IC) within CIS lesions. * *p* < 0.05; ** *p* < 0.01; *** *p* < 0.001.

Significant inverse correlations were observed between PD-L1 and GATA3 expression in TC within MIBC (rs = −0.661, *p* = 0.009) as well as in CIS (rs = −0.607, *p* = 0.024). In addition, ADAM17 positively correlated with IL-10R in IC within CIS (Figure 4). Expression of ADAM17 showed a trend toward inverse correlation with PD-L1 within both tumor regions and cell types, but without statistically significant differences (TC-MIBC: rs = −0.413, *p* = 0.170; TC-CIS: rs = −0.198, *p* = 0.486; IC-MIBC: rs = −0.348, *p* = 0.215; IC-CIS: rs = −0.365, *p* = 0.184).

### 3.4. ADAM10 Expression Is Associated with Advanced Tumor Disease at RC

**Figure 5:**
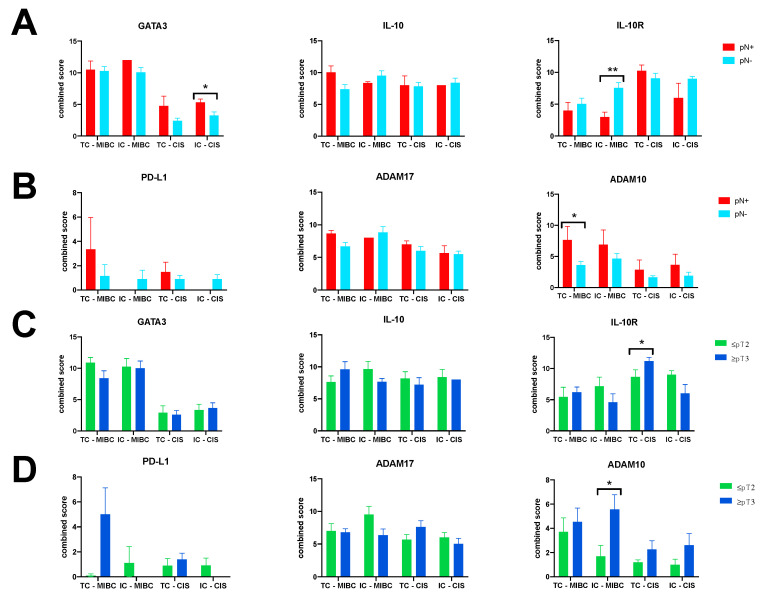
Total expression levels of GATA3, PD-L1, IL-10, and IL-10R, and ADAM17/10 proteases depending on lymph node status (**A**,**B**) and tumor stage (**C**,**D**) on RC specimens. Data represent mean ± SEM (* *p* < 0.05; ** *p* < 0.01; *** *p* < 0.001).

With regard to PD-L1, IL-10, and ADAM17, no significant association was noticed between combined IHC scores and occurrence of extravesical tumor disease and positive pathological lymph node disease at RC (Figure 5A–D). However, ADAM10 expression seems to be associated with advanced tumor disease. In detail, expression of ADAM10 (mean combined score ± SD) was consistently higher in RC specimens with pN+ status and extravesical disease (Figure 5A,D). Significant differences for mean combined scores concerning ADAM10 expression were identified in TC within MIBC for lymph node status (7.67 ± 4.9 vs. 3.6 ± 2.2; *p* = 0.020), and in IC within MIBC for extravesical disease (5.6 ± 3.6 vs. 1.7 ± 2; *p* = 0.049). For IL-10R, discordant results depending on cell type and tumor region were noticed for lymph node status and tumor disease (Figure 5A,C). GATA3 expression was homogeneously expressed in both cell types and tumor regions for lymph node status as well as tumor stage. Significantly higher GATA3 expression levels were assessed in IC within CIS for lymph node disease (5.3 ± 1.1 vs. 3.3 ± 1.9; *p* = 0.044), (Figure 5A,C).

## 4. Discussion

Th1 type cell-mediated immunity is required for BCG response [22,23,24,25]. However, immune checkpoints such as PD-1/PD-L1 can prevent the development of Th1 type immunity [26]. In support of this concept, Kates et al. recently reported that baseline expression of PD-L1 in 25–28% of bladder cancer patients correlated with BCG failure [11]. In the present study, we detected PD-L1 expression in only 4 (21.1%) of 19 patients with BCG failure (MIBC and CIS). Moreover, we found that the absence of PD-L1 was associated with strong expression of ADAM17 and, although less pronounced, with expression of ADAM10 within both bladder tumor regions. This is intriguing because ADAM10 and ADAM17 have recently been shown to cleave PD-L1 from the surface of cancer cells [18]. The resulting soluble PD-L1 fragment induced apoptosis in cytotoxic CD8+ T cells and thus compromised T cell-mediated tumor cell killing. Collectively, these findings raise the possibility that ADAM proteases cleave PD-L1 in bladder cancer tissues and thus contribute to immune suppression and BCG resistance (Supplemental Figure S2).

ADAM proteases are multi-domain ectoenzymes residing at the cell surface, where they proteolytically cleave adjacent membrane-anchored proteins and thus convert them into soluble forms [27]. This process, also known as ectodomain shedding, may be particularly important in the context of tumor immunotherapy because ADAM proteins regulate both tumor cell biology [28] and immune cell function [29]. Among the 21 family members in humans, ADAM10 and ADAM17 have been examined in most detail.

ADAM sheddase activity is exemplified by the cleavage of membrane-bound tumor necrosis factor-α (TNF-α) by ADAM17, which was therefore initially referred to as TNF-α converting enzyme (TACE). ADAM17 can also release the receptors for TNF-α, sTNFR1, and sTNFR2. Likewise, other cytokines and their receptors have also been shown to be substrates of ADAM proteases, indicating that ADAM-mediated ectodomain shedding is critical in immune activation and regulation.

Another important function of ADAM17 relates to epidermal growth factor receptor (EGFR) activation. In addition to exogenous EGF, EGFR can also be activated by endogenous ligands such as heparin-binding EGF (HB-EGF), amphiregulin (AREG), and transforming growth factor-α (TGF-α), all of which can be released from the plasma membrane by ADAM17 [30].

Moreover, ADAM-dependent release of endogenous EGFR ligands has been described as a key step in EGFR transactivation by G protein-coupled receptors [31]. In cancer, several ADAM proteases promote malignancy by EGFR transactivation (Supplemental Figure S2), supporting epithelial to mesenchymal transition (EMT) via cleavage of E-cadherin. In addition to being a dominant feature of cancer biology, EMT endows many cell types with migratory and invasive capacity [32]. Along this line, EMT has been suggested to be a prerequisite for muscle invasion/metastasis in bladder cancer [33], although the role of ADAMs in BCG-refractory bladder cancer has not been addressed in this study. Additionally, ADAM17 was shown to be enhanced by platinum-based chemotherapy and caused EGFR transactivation by stimulating AREG release [34]. In this context, ADAM17 inhibition sensitized cancer cells to cisplatin-induced apoptosis.

In our study, we could also show that expression of ADAM10, which is the principal sheddase in Notch signaling and regulates cell fate and differentiation, was associated with advanced tumor disease at RC, resulting in a higher incidence of positive pathological lymph node and extravesical disease. Our observation is in accordance with previous work reporting that ADAM10 regulates proliferation, invasion, and chemoresistance of bladder cancer cells [35]. Thus, ADAM analysis may be attractive to circulating tumor cells in M+ patients. In addition to our findings related to ADAM proteases and PD-L1, we obtained evidence for a generally immunosuppressive milieu in bladder cancer tissue from patients with BCG resistance. IL-10 expression was consistently increased in both tumor regions and cell types, whereas IL-10R was differentially expressed, with higher levels in IC compared to TC.

We have previously reported that expression of the transcription factor GATA3 is high in bladder cancer tissue prior to the onset of BCG therapy [10,36]. Here, we extend these findings and show that GATA3 expression remained high in BCG-resistant bladder cancer tissue (MIBC and CIS lesions). This is of interest because strong GATA3 expression has already been confirmed as an independent predictor of poor prognosis in MIBC [37].

The limitations of our study include the small and heterogeneous (including isolated CIS lesions as well as papillary recurrences) patient collective and the retrospective study design, which limits statistical power. Additionally, our preliminary findings are only hypothesis-generating, describing, for the first time, the expression of ADAM proteases and the lack of PD-L1 in BCG failure. Moreover, mechanistic proof for ADAM17-mediated PD-L1 release is lacking. In addition, no control group (BCG responders) was included, so we have no head-to-head comparison of ADAM expression levels between BCG responders and BCG failure. Our findings, however, imply that IHC assessment of PD-L1 expression may not be sufficient and should be supplemented by measurement of ADAM17 and possibly also of PD-L1 in patients with BCG failure. Protein ectodomains shedded from the cell surface by metalloproteases are indeed gaining interest as soluble cancer biomarkers, also in predicting response to checkpoint inhibitors [38,39,40]. Additional studies are certainly needed to determine whether ADAM17 IHC will improve the prediction of clinical outcomes in bladder cancer therapy, although TCGA (The Cancer Genome Atlas) data provide the first evidence that ADAM17 mRNA expression alone is a significant prognostic indicator of survival in bladder cancer (Supplemental Figure S3) [41,42].

Our data further suggest that ADAM17 may be involved in PD-L1 inhibitor resistance [18]. We therefore believe that our findings should be considered when it comes to the interpretation of data from clinical trials combining BCG with checkpoint inhibitors in BCG failure. Moreover, ADAM proteases may even drive the process of muscle invasion of bladder cancer, as they have been implicated in cell migration [27]. ADAM proteases may thus not only be relevant for diagnostic purposes but may also serve as attractive targets in the treatment and follow-up of bladder cancer to improve personalized medicine, although their validation and implementation into clinical routine will be challenging [38].

In conclusion, the role of ADAM proteases in the biology and immunotherapy of urologic tumors has received little attention so far. Here, we observed strong ADAM17 and ADAM10 expression associated with the lack of PD-L1 expression in RC specimens obtained from patients with BCG failure. ADAM protease expression was associated with the lack of PD-L1 expression, giving rise to a scenario in which ADAMs may cleave PD-L1 from the surface of bladder TC and possibly also from IC. Moreover, ADAM10 was linked with advanced tumor stage and positive lymph node status at RC. Collectively, our findings suggest that ADAM proteases may be attractive diagnostic as well as therapeutic targets in bladder cancer in general, and especially during BCG-based immunotherapy of non-muscle-invasive bladder cancer. In addition, our work seems to have implications for current concepts that combine BCG with checkpoint inhibitors. Accordingly, we observed strong ADAM 17 expression associated with the lack of PD-L1 expression in RC specimens obtained from patients with BCG failure.

## Figures and Tables

**Table 1:** Semi-quantitative IHC analysis.

**Staining Intensity Score (0–3)**	
0	No staining
1	Weak staining
2	Moderate staining
3	Intense staining
**Percentage of stained cells score (0–4)**	
0	0%
1	1–4%
2	5–9%
3	10–49%
4	≥50%
**Combination Score = % score (0–4) x intensity score (0–3)**	
negative	0–3
positive	≥4

A modified quick score was used for semi-quantification of tumor cells (TC) and immune cells (IC) within the two different tumor regions (MIBC and carcinoma in situ). The product of both scores (intensity and proportion) was used to indicate the level of protein expression. Protein expression was considered negative when the combination IHC score was ≤3 and positive when 4 or greater.

**Table 2:** Overview of histopathological features concerning primary transurethral resection of the bladder (TURB) (prior Bacillus Calmette Guérin (BCG) induction), TURB at BCG failure, and finally, histopathology in RC specimens.

	Primary TURB (Prior BCG Induction)	TURB at BCG Failure	RC Specimens
Primary CIS, *n* (%)	3 (15.8%)	3 (15.8%)	5 (26.3%)
Concurrent CIS, *n* (%)	9 (47.4%)	10 (52.6%)	10 (52.6%)
pTa, *n* (%)	7 (36.8%)	-	-
pT1, *n* (%)	9 (47.4%)	8 (42.1%)	-
pT2a, *n* (%)	-	8 (42.1%)	1 (5.3%)
pT2b, *n* (%)	-	-	4 (21.1%)
pT3a, *n* (%)	-	-	4 (21.1%)
pT3b, *n* (%)	-	-	5 (26.2%)
High grade (HG), *n* (%)	19 (100%)	19 (100%)	19 (100%)
Low grade (LG)	-	-	-
pN+ status, *n* (%)			5 (26.3%)

## Data Availability

The data presented in the study are available on request from the corresponding author. The data are not publicly available due to privacy.

## Supplementary Materials

The following are available online at https://www.mdpi.com/2077-0383/10/4/764/s1, Figure S1: (A) Representative IHC images of IL-10 and its receptor (IL-10R) within MIBC in three patients with PD-L1 negative status (#1-3, pure urothelial carcinoma, comb. score of 0 in all patients) and one patient (#4) with positive PD-L1 expression (combined score of 12, urothelial carcinoma with squamous differentiation). (B) Mean IHC combined scores for IL-10 and IL-10R assessed for both tumor regions (MIBC and CIS) and both cell types (tumor cells and immune cells) were shown. Statistically significant differences in expression patterns were confirmed for IL-10R. Data represent mean ± SEM (* *p* < 0.05; ** *p* < 0.01; *** *p* < 0.001 according to independent samples Kruskal–Wallis test and Mann–Whitney U test). Figure S2. A schematic model of ADAM proteases-induced PD-L1 cleavage from the surface of bladder tumour cells and possibly also immune cells. PD-L1 cleavage produces soluble PD-L1 (sPD-L1), which may directly induce CD8+ T cell apoptosis one the one hand, and on the other compete with PD-(L)1 inhibitors [1]. Moreover, several ADAMs promote malignancy by stimulating cell proliferation via epidermal growth factor receptor (EGFR) transactivation and by the induction of epithelial–mesenchymal transition (EMT) via cleavage of E-cadherin [2–4]. Adapted with permission from ref. [5]. 2021 nature reviews urology. ADAM: a disintegrin and metalloproteinase. Kaplan Meier survival analysis concerning ADAM17 mRNA expression in bladder cancer extracted from TGCA data. High ADAM17 mRNA expression is significantly associated with poor survival compared with low mRNA expression levels. Based on the current cut off of 9.5 FKPM from TGCA data, 5-year survival rate is 45% (low expression) versus 28% (high expression) [6,7].

## Abbreviations

ADAM	a disintegrin and metalloproteinase
BCG	Bacillus Calmette Guérin
CIS	carcinoma in situ
HG	high grade
IC	immune cells
IHC	immunohistochemistry
IL	interleukin
n.s.	not significant
NMIBC	non-muscle-invasive bladder cancer
MIBC	muscle-invasive bladder cancer
PD-L1	programmed death ligand 1
RC	radical cystectomy
TC	tumor cells
TURB	transurethral resection of the bladder
