# Supplementary material for: Expression of ADAM Proteases in Bladder Cancer Patients with BCG Failure: A Pilot Study
Source: J Clin Med. 2021 Feb 14;10(4):764. doi: 10.3390/jcm10040764 (PMC7917772; doi:10.3390/jcm10040764)
Supplement: Supplementary file 1 [file jcm-10-00764-s001.pdf]

Supplementary Material

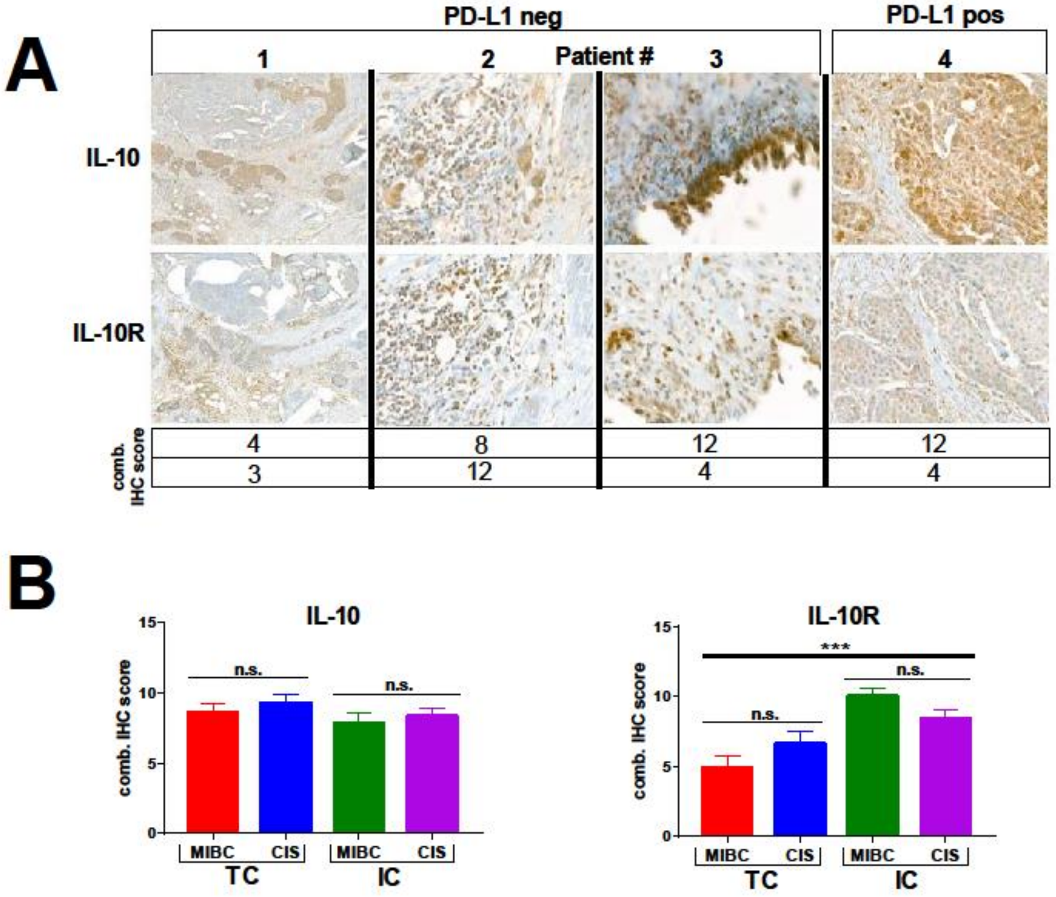

Figure S1. **(A)** Representative IHC images of IL-10 and its receptor (IL-10R) within MIBC in 3 patients with PD-L1 negative status (#1–3, pure urothelial carcinoma, comb. score of 0 in all patients) and one patient (#4) with positive PD-L1 expression (combined score of 12, urothelial carcinoma with squamous differentiation). **(B)** Mean IHC combined scores for IL-10 and IL-10R assessed for both tumor regions (MIBC and CIS) and both cell types (tumour cells and immune cells) are shown. Statistically significant differences of expression patterns were confirmed for IL-10R. Data represent mean ± SEM (*$p < 0.05$; **$p < 0.01$; ***$p < 0.001$ according to independent-samples Kruskal-Wallis test and Mann-Whitney *U* test). IC: immune cells; TC: tumor cells; CIS: concurrent carcinoma in situ; MIBC: muscle-invasive bladder cancer; IHC: immunohistochemical.

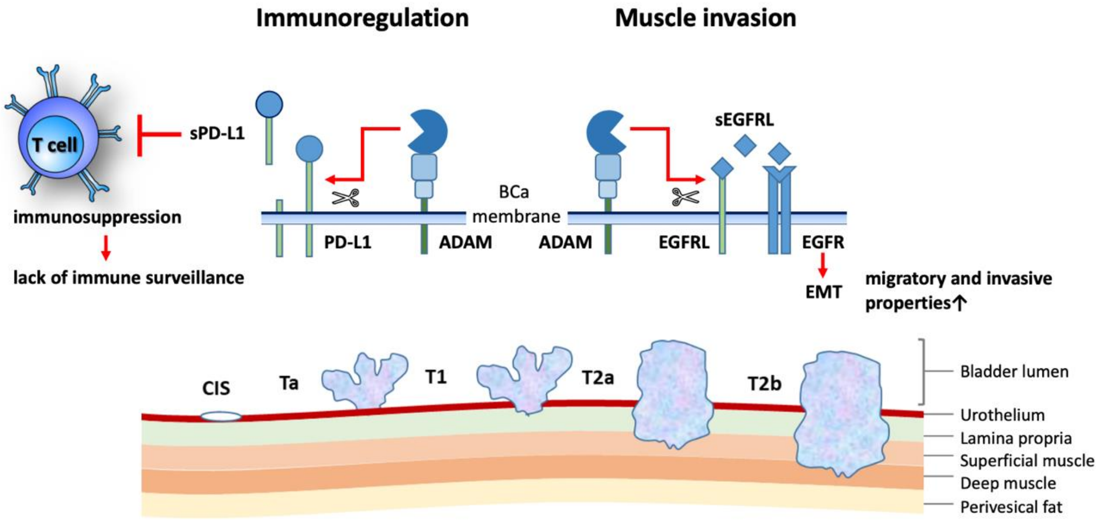

Figure S2. A schematic model of ADAM proteases-induced PD-L1 cleavage from the surface of bladder tumour cells and possibly also immune cells. PD-L1 cleavage produces soluble PD-L1 (sPD-L1), which may directly induce CD8+ T cell apoptosis one the one hand, and on the other compete with PD-(L)1 inhibitors [1]. Moreover, several ADAMs promote malignancy by stimulating cell proliferation via epidermal growth factor receptor (EGFR) transactivation and by the induction of epithelial–mesenchymal transition (EMT) via cleavage of E-cadherin [2–4]. Adapted with permission from ref. [5]. 2021 nature reviews urology. ADAM: a disintegrin and metalloproteinase.

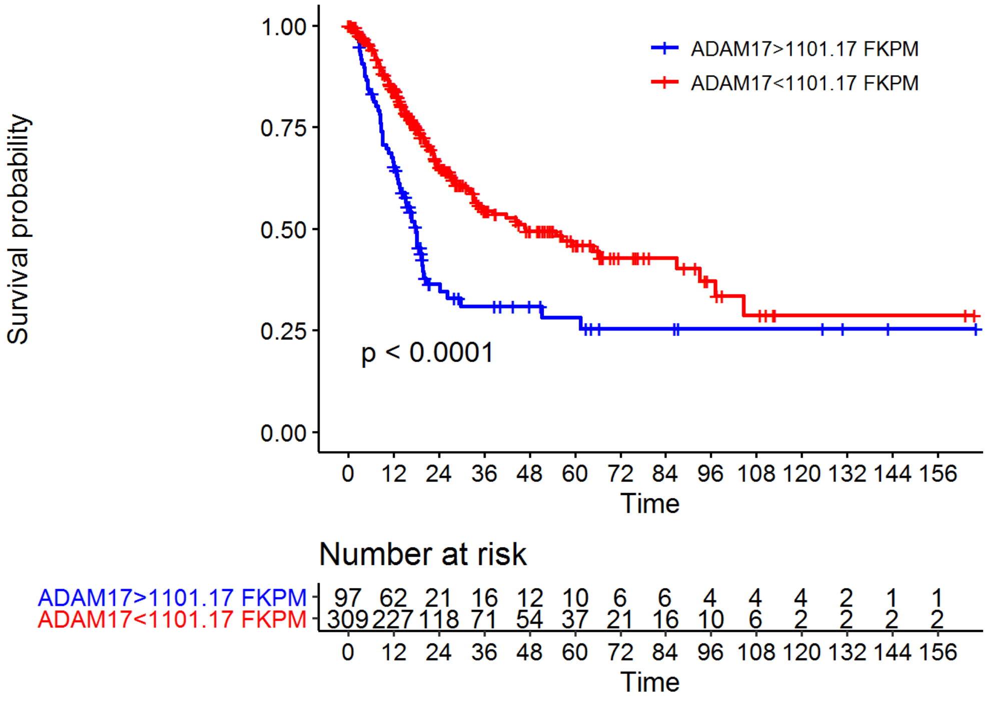

Figure S3. Kaplan Meier survival analysis concerning ADAM17 mRNA expression in bladder cancer extracted from TGCA data. High ADAM17 mRNA expression is significantly associated with poor survival compared with low mRNA expression levels. Based on the current cut off of 9.5 FKPM from TGCA data, 5-year survival rate is 45% (low expression) versus 28% (high expression) [6,7].

References

1. Orme, J.J.; Jazieh, K.A.; Xie, T.; Harrington, S.; Liu, X.; Ball, M.; Madden, B.; Charlesworth, M.C.; Azam, T.U.; Lucien, F.; et al. ADAM10 and ADAM17 cleave PD-L1 to mediate PD-(L)1 inhibitor resistance. *Oncoimmunology* **2020**, *9*, 1744980, doi:10.1080/2162402X.2020.1744980.
2. Prenzel, N.; Zwick, E.; Daub, H.; Leserer, M.; Abraham, R.; Wallasch, C.; Ullrich, A. EGF receptor transactivation by G-protein-coupled receptors requires metalloproteinase cleavage of proHB-EGF. *Nature* **1999**, *402*, 884–888, doi:10.1038/47260.
3. Thiery, J.P.; Acloque, H.; Huang, R.Y.; Nieto, M.A. Epithelial-mesenchymal transitions in development and disease. *Cell*. **2009**, *139*, 871–890, doi:10.1016/j.cell.2009.11.007.
4. McConkey, D.J.; Choi, W.; Marquis, L.; Martin, F.; Williams, M.B.; Shah, J.; Svatek, R.; Das, A.; Adam, L.; Kamat, A.; et al. Role of epithelial-to-mesenchymal transition (EMT) in drug sensitivity and metastasis in bladder cancer. *Cancer Metastasis Rev*. **2009**, *28*, 335–344, doi:10.1007/s10555-009-9194-7.
5. Jordan, B.; Meeks, J.J. T1 bladder cancer: Current considerations for diagnosis and management. *Nat. Rev. Urol*. **2019**, *16*, 23–34, doi:10.1038/s41585-018-0105-y.
6. Uhlen, M.; Zhang, C.; Lee, S.; Sjöstedt, E.; Fagerberg, L.; Bidkhori, G.; Benfeitas, R.; Arif, M.; Liu, Z.; Edfors, F.; et al. A pathology atlas of the human cancer transcriptome. *Science* **2017**, *357*, eaan2507, doi:10.1126/science.aan2507.
7. Available online: https://www.proteinatlas.org/ENSG00000151694-ADAM17/pathology/urothelial+cancer (accessed on 5 Nov 2020).
